# Fluorescent cystoscopy-assisted en bloc transurethral resection versus conventional transurethral resection in patients with non-muscle invasive bladder cancer: study protocol of a prospective, open-label, randomized control trial (the FLEBER study)

**DOI:** 10.1186/s13063-021-05094-y

**Published:** 2021-02-12

**Authors:** Makito Miyake, Nobutaka Nishimura, Takashi Inoue, Shota Suzuki, Tomomi Fujii, Takuya Owari, Shunta Hori, Yasushi Nakai, Michihiro Toritsuka, Hitoshi Nakagawa, Shinji Tsukamoto, Satoshi Anai, Kazumasa Torimoto, Tatsuo Yoneda, Nobumichi Tanaka, Kiyohide Fujimoto

**Affiliations:** 1 Department of Urology, Nara Medical University, 840 Shijo-cho, Kashihara, Nara, 634-8522 Japan; 2 Institute for Clinical and Translational Science, Nara Medical University, 840 Shijo-cho, Kashihara, Nara, 634-8522 Japan; 3 Department of Diagnostic Pathology, Nara Medical University, 840 Shijo-cho, Kashihara, Nara, 634-8522 Japan; 4 Department of Psychiatry, Nara Medical University, 840 Shijo-cho, Kashihara, Nara, 634-8522 Japan; 5 Cardiovascular Medicine, Nara Medical University, 840 Shijo-cho, Kashihara, Nara, 634-8522 Japan; 6 Department of Orthopedic Surgery, Nara Medical University, 840 Shijo-cho, Kashihara, Nara, 634-8522 Japan; 7 Department of Prostate Brachytherapy, Nara Medical University, 840 Shijo-cho, Kashihara, Nara, 634-8522 Japan

**Keywords:** Non-muscle invasive bladder cancer, Transurethral resection, En bloc resection, Recurrence, 5-aminolevulinic acid, Fluorescent cystoscopy, Photodynamic diagnosis, Randomized controlled trial

## Abstract

**Background:**

Transurethral resection of bladder tumor (TURBT) is an essential procedure both for the treatment and staging of bladder cancer, particularly non-muscle invasive bladder cancer (NMIBC). The dissemination of cancer cells during resection and the consequent seeding into the bladder mucosa is the main cause of post-TURBT intravesical recurrence. Although the tumor dissemination is inevitable during conventional TURBT (cTURBT), this drawback can be overcome by tumor resection in one piece with intact surrounding normal tissues, referred to as en bloc resection. We previously described the photodynamic diagnosis (PDD)-assisted en bloc TURBT (EBTUR) technique and its favorable outcomes. Based on our preliminary studies, this randomized controlled trial was designed to evaluate the superiority of PDD-EBTUR to PDD-cTURBT.

**Methods:**

The FLEBER study is a single-center randomized controlled trial in NMIBC patients who require TURBT. The longest diameter of the tumor must be between 6 and 30 mm. A total of 160 eligible patients will be enrolled after screening and randomly allocated to the PDD-EBTUR (experimental) and PDD-cTURBT (control) groups in a 1:1 ratio (80 cases to 80 cases). All patients will be treated using a single, immediate postoperative intravesical chemotherapy with epirubicin. The primary endpoint of this trial is the 2-year recurrence-free survival after surgery in pathologically proven low- or intermediate-risk NMIBC. All patients will be monitored by cystoscopy and urine cytology every 3 months for 2 years. Patient data including adverse events and complications, and data from frequency volume charts, pain scales, and health-related QOL questionnaires will be collected before and after the TURBT at indicated visits.

**Discussion:**

The goal of this trial is to determine the potential benefits of PDD-cTURBT and PDD-EBTUR followed by a single immediate postoperative intravesical chemotherapy in patients with low- or intermediate-risk NMIBC who undergo TURBT. Ultimately, our findings will lead to the development of better interventions and potentially change the standard of care.

**Trial registration:**

This clinical trial was prospectively registered with the UMIN Clinical Trials Registry on 1 August 2020. The reference number is UMIN000041273, and the Ethics Committee of Nara Medical University Approval ID is 2702.

**Supplementary Information:**

The online version contains supplementary material available at 10.1186/s13063-021-05094-y.

## Background

Transurethral resection of bladder tumor (TURBT) is an essential procedure both for the treatment and staging of bladder cancer, particularly non-muscle invasive bladder cancer (NMIBC) [1]. Residual tumors and overlooked tumors directly lead to underdiagnosis and intravesical recurrence after the initial TURBT. Several randomized controlled trials (RCTs) confirmed that fluorescent cystoscopy using 5-aminolevulic acid (ALA) or its hexyl ester, hexaminolevulinate, could improve tumor detection rate and recurrence-free survival (RFS) compared to white-light cystoscopy alone [2, 3]. Moreover, the additionally detected lesions through photodynamic diagnosis (PDD) significantly modified the recurrence- and progression-risk categories and led to the recommendation of postoperative treatment compared to white-light cystoscopy alone (19% vs. 6.3%) [4]. In addition to this evidence, two pivotal studies in Japan provided favorable evidence for PDD-assisted TURBT (PDD-TURBT). As a consequence, the latest Japanese clinical practice guidelines 2019 recommended the use of PDD-TURBT for the treatment of NMIBC [5].

Intravesical recurrence after TURBT is largely attributed to the dissemination of cancer cells and the consequent seeding into the bladder mucosa [6]. This dissemination during resection is inevitable during conventional TURBT because bladder tumors need to be cut into fragments and taken out from the bladder piecemeal. This drawback can be overcome through tumor resection in one piece with intact surrounding normal tissues. Since the first report by Kawada et al. in 1997, the usefulness and safety of en bloc TURBT (EBTUR) has been reported by several publications, systematic reviews, and meta-analyses [7–15]. An RCT conducted by Chen et al. demonstrated that EBTUR using two-micron continuous-wave Tm:YAG laser did not reduce the risk of recurrence compared to conventional TURBT [10]. However, transurethral laser therapy is not widespread yet, especially in Asian countries, with the exception of China [12, 13, 15, 16]. Moreover, previous studies did not involve PDD visualization of tumors, which is currently recommended for the treatment of NMIBC [5]. We recently reported our initial experience involving the use of PDD-en bloc TURBT (PDD-EBTUR). We noted that PDD helped significantly during the circumferent demarcation of the tumor [15]. None of the 12 patients in the PDD-EBTUR group experienced intravesical recurrence, while two of the 29 patients in the historical control PDD-conventional TURBT (PDD-cTURBT) group experienced Ta low-grade recurrent tumors [15].

Currently, very few well-designed RCTs have compared the clinical values of EBTUR and cTURBT. This prospective, open-label, single-center, randomized control trial (the FLEBER study) will evaluate the oncological outcomes, postoperative complications, lower urinary symptoms, and the changes in health-related quality of life (HR-QOL) following PDD-EBTUR. To the best of our knowledge, this is the first study to compare the clinical benefits of oral PDD-EBTUR and PDD-cTURBT.

## Methods/design

### Registration

This trial will be conducted in accordance with the principles of the Declaration of Helsinki (64th WMA General Assembly, Fortaleza, Brazil, October 2013 version). The study protocol was approved by the Ethics Committee of Nara Medical University (Approval ID: 2702) and this trial was registered in the UMIN Clinical Trial Registry on 1 August 2020. (ID: UMIN000041273) before patient recruitment. The URL of the trial registry record is https://upload.umin.ac.jp/cgi-open-bin/ctr/ctr_view.cgi?recptno=R000047138.

### Patient recruitment, inclusion criteria, exclusion criteria, and study design

**Fig. 1:**
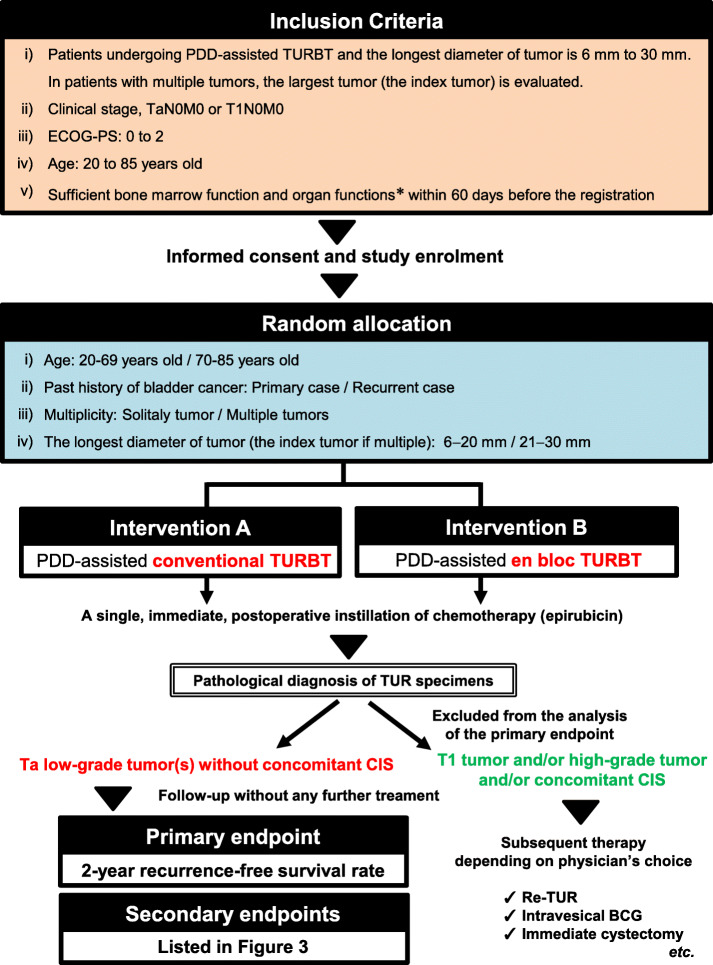
Design of the FLEBER study: inclusion criteria and endpoints. Patients should fulfill the indicated inclusion criteria to be eligible for this trial. *Hemoglobin, ≥ 9.0 g/dL; white blood cell count, ≥ 12,000/mm^3^; absolute neutrophil count, ≥ 2000 cells/mm^3^; platelet count, ≥ 100,000 cells/mm^3^; normal kidney and liver functions as determined by creatinine, total bilirubin, aspartate transaminase (AST), and alanine transaminase (ALT) ≤ 2 × the upper limit of normal (ULN) for the reference laboratory. Abbreviations: PDD, photodynamic diagnosis; TURBT, transurethral resection of bladder tumor; ECOG-PS, Eastern Cooperative Oncology Group-Performance Status; CIS, carcinoma in situ; BCG, Bacillus Calmette-Guérin

**Fig. 2:**
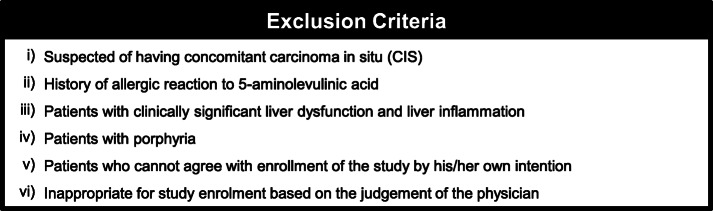
Exclusion criteria for the FLEBER study

This prospective, open-label, single-center trial in patients with localized NMIBC is ongoing at the Nara Medical University (a Japanese academic hospital). The trial design and protocol adhere to the Recommendations for Interventional Trials (SPIRIT) criteria (see Additional file 1). The completed SPIRIT checklist is provided in the Supplementary materials section. This RCT is the parallel group, two-arm, superiority trial with 1:1 allocation ratio. The flowchart, inclusion criteria, and study design are shown in Fig. 1. The exclusion criteria are listed in Fig. 2. Informed consent and written consent forms were obtained from patients before study enrollment. The consent form and explanatory documents given to participants is provided in the Supplementary materials (Additional files 2 and 3), which describes the provided information including surgical procedures, PDD-cTURBT and PDD-EBTUR. After agreement to participate in the clinical trial, participants will be randomized with a 1:1 allocation into PDD-EBTUR and PDD-cTURBT treatment arms (permuted block method) using the web-based system of Mujinwari (Iruka System Ltd., Tokyo, Japan), which is based on four factors: age, past history of NMIBC, multiplicity, and the longest diameter of the tumor. YN generates the allocation sequence, and MM will enroll participants and assign participants to interventions. Surgeons and patients will be informed to which treatment arm the patients is allocated: PDD-cTURBT arm or PDD-EBTUR arm. If participants refuse the allocated surgical procedure, the procedure will be changed according to the participant request. The participant will be considered a drop-out case.

Patients with tumors less than 6 mm in diameter will be excluded from this trial because small tumors can be resected with a single cut using a standard loop electrode. In addition, patients with tumors more than 30 mm in diameter will be excluded because large tumors cannot be removed en bloc from the bladder using a 28-Fr TUR resectoscope sheath. Patients who are suspected of having carcinoma in situ (CIS) will be excluded because these patients will have to be treated using intravesical Bacillus Calmette-Guérin (BCG) postoperatively, and the carcinoma cannot be resected completely using EBTUR.

### Surgical procedure and device for PDD-EBTUR and PDD-cTURBT

Approximately 3 h (range, 2–4 h) before surgery, patients orally received a water-dissolved 5-ALA solution at a dose of 20 mg/kg (ALAGLIO®; Chugai Pharmaceutical Co., Ltd., and SBI Pharmaceuticals Co., Ltd.). The PDD-EBTUR will be performed by a single surgeon with substantial experience in this surgical technique (M. Miyake), while the PDD-cTURBT will be performed by one of the following experienced urologists: M. Miyake, S. Hori, Y. Nakai, S. Anai, K. Torimoto, N. Tanaka, and K.Fujimoto. The surgical procedures and devices for PDD-EBTUR and PDD-cTURBT are described in our previous report [15].

### A single immediate postoperative intravesical instillation of chemotherapy (IPIC)

All participants will undergo IPIC unless bladder perforation occurs or is suspected during TUR. Within 24 h postoperatively, one intravesical instillation of 60 mg of epirubicin in 30 ml of saline will be administered. The catheter will be clamped and left for 1 h, and subsequently unclamped. The patients who do not undergo IPIC will be excluded from the analysis of primary endpoint, but included in the analysis of secondary endpoints.

### Follow-up, data collection, and data protection

**Fig. 3:**
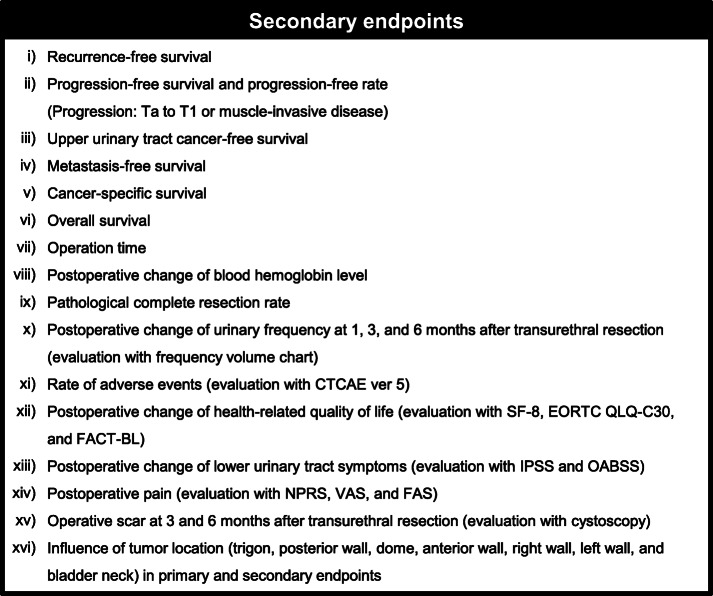
Secondary endpoints of the FLEBER study. Abbreviations: CTCAE, Common Terminology Criteria for Adverse Events; SF-8, Short Form-8 health survey; EORTC, The European Organization for Research and Treatment of Cancer; FACT-BL, Functional Assessment of Cancer Therapy-Bladder; NPRS, numerical pain rating scale; VAS, visual analog scale; FPS, faces pain scale; IPSS, International Prostate Symptom Score; OABSS, overactive bladder symptom score

The recently updated guidelines of the Japanese Association of Urology and European Association of Urology will be used to stratify patients with NMIBC into low-, intermediate-, high-, and highest-risk groups [5, 17]. According to evidence obtained from real-world data, IPIC could reduce the risk of intravesical recurrence in both low-risk and intermediate-risk NMIBC patients [18]. In this trial, patients with low-risk and intermediate-risk NMIBC will be followed up without any further adjuvant treatment. In contrast, patients with high- or highest-risk NMIBC based on TURBT specimens should be managed by extensive treatment such as re-TUR, intravesical BCG, and immediate cystectomy at the discretion of the physicians. They will be excluded from the analysis of the primary endpoint, but included in the analysis of the secondary endpoints (Fig. 3).

**Fig. 4:**
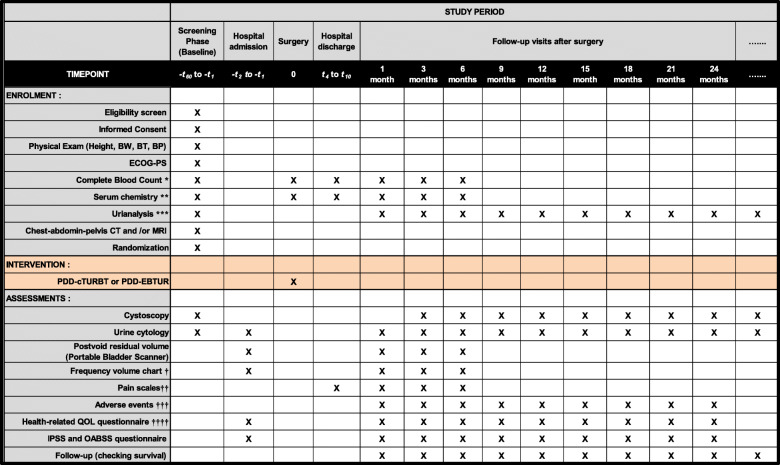
Intervention and assessment schedule for the FLEBER study according to the Recommendations for Interventional Trials (SPIRIT). Follow-up visits and data collection should occur approximately 1, 3, 6, 9, 12, 15, 18, 21, and 24 months from the surgery. Patients will complete a set of questionnaires at every visit, and follow-up information may be collected via medical charts. The Case Report Form will include information regarding past history, concomitant medications, and any medications taken after the treatment. Chest–abdomen–pelvis computed tomography (CT) and/or magnetic resonance imaging (MRI) should be performed for TNM classification. X, mandatory; *hemoglobin, hematocrit, white blood cell count and fractions, platelet count; **aspartate transaminase (AST), alanine transaminase (ALT), γ-glutamyl transpeptidase (γ-GTP), total bilirubin, alkaline phosphatase (ALP), lactate dehydrogenase (LDH), total protein, albumin, serum creatinine, uric acid, total cholesterol, low-density lipoprotein (LDL)-cholesterol, high-density lipoprotein (HDL)-cholesterol, triglyceride, C-reactive protein (CRP), calcium, phosphorus, potassium, chloride; ***Urine dipstick test (specific gravity, pH, protein, glucose, bilirubin, urobilinogen, ketone body, and occult blood) and urine sediment test; ^†^3 days and nights record. Patients pick days that will be convenient for them to measure and record everything; ^††^pain scale measures: numerical pain rating scale (NPRS), visual analog scale (VAS), and faces pain scale (FPS); ^†††^according to the Common Toxicity Criteria for Adverse Events (CTCAE v 5.0) translated into Japanese; ^††††^questionnaires: SF-8™, EORTC QLQ-C30, and FACT-BL. Abbreviations: BW, body weight; BT, body temperature; BP, blood pressure; ECOG-PS, Eastern Cooperative Oncology Group-performance status scale; PDD, photodynamic diagnosis; EBTUR, en bloc transurethral resection of bladder tumor; cTURBT, conventional transurethral resection of bladder tumor; QOL, quality of life; IPSS, International Prostate Symptom Score; OABSS, overactive bladder symptom score

Patient data will be collected before the surgery, and 1, 3, 6, 9, 12, 15, 18, 21, and 24 months after the surgery (Fig. 4). Recurrence was defined as pathologically proven urothelial carcinoma. Progression was defined as a recurrent disease with upstaging from Ta to T1 or invasion into the muscularis propria (≥ T2), positive regional lymph node, and/or distant metastases. After 24 months of follow-up, patients will be followed-up every 6 months for 3 years, and annually thereafter. To improve monitoring adherence, the clinician will take time to explain about need for post-operative surveillance examination and encourage the participants to undergo routine cystoscopy and urine cytological examination.

One, 3, and 6 months after the surgery, the 24-h urinary frequency, total voided volume, and mean voided volume will be recorded on a frequency volume chart for 3 days [19]. Postoperative pain intensity will be assessed using the numerical pain rating scale, visual analog scale, and faces pain scale. The assessment includes patient-reported outcomes (PROs) such as the SF-8™, EORTC QLQ-C30, FACT-BL, International Prostate Symptom Score (IPSS), and overactive bladder symptom score (OABSS). The postoperative observational evaluation such as PROs can be discontinued upon participant request. The data will be documented in specific Case Report Forms for genitourinary adverse events (AEs) and other possible AEs using the Common Toxicity Criteria for Adverse Events (CTCAE v 5.0). AEs strongly related to the intervention of PDD-cTURBT or PDD-EBTUR are listed as follows: “Intraoperative urinary injury,” “Cystitis noninfective,” “Urinary tract infection,” “Dysuria,” “Hematuria,” “Bladder perforation,” “Urinary frequency,” “Urinary incontinence,” “Urinary retention,” “Urinary tract pain,” and “Urinary urgency.” Any other potential AEs are recorded and grouped into “related AEs” or “unrelated AEs.” When serious AEs occur, investigators report to the Safety Monitoring Committee.

Yoshiaki Matsumura and Yoshitaka Itami (Nara Prefecture General Medical Center) are members of data management team. They are not involved in participant-facing elements of the clinical trial. They are responsible for the data entry from medical charts, data security, and storage, including any related processes to promote data quality such as data double-checking to central data monitoring. To protect the patient personal information, unique identification codes (FLEBER IDs) will be assigned to all patients. All the data will be protected in password-accessible electronic data files on secure servers before, during, and after the trial, and only investigators will be able to access the files. All data and documents will be deleted and discarded 5 years after the end of the trial, unless the data are being used for another study.

### Determining the target sample size

**Fig. 5:**
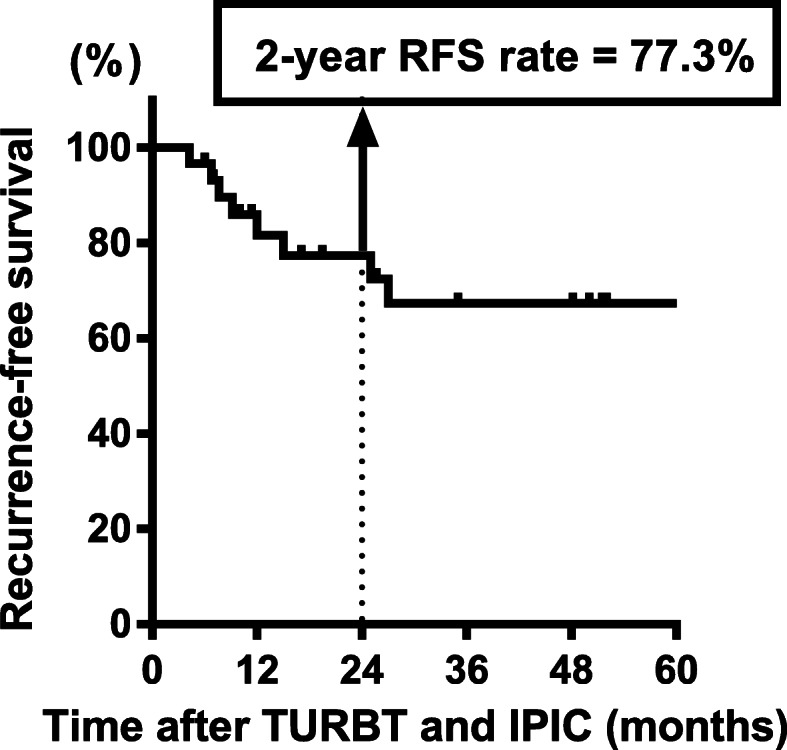
Recurrence-free survival curve after TURBT and immediate postoperative intravesical chemotherapy in patients with low- or intermediate-risk NMIBC. The analysis was based on our historical dataset, which included data from more than 1500 patients with NMIBC. Thirty-four patients were included in order to obtain the recurrence-free survival curve. Abbreviations: RFS, recurrence-free survival; TURBT, transurethral resection of bladder tumor; IPIC, immediate postoperative intravesical chemotherapy; NMIBC, non-muscle invasive bladder cancer

To date, we have conducted several observational studies on the oncological outcomes of NMIBC patients treated using TURBT with or without intravesical treatment [1, 20–23]. Our study subjects include patients with low- or intermediate-risk NMIBC, who were treated with TURBT followed by IPIC (with no further adjuvant treatment) and who were eligible for the pilot analysis. We observed that the 2-year RFS rate in this subset was 77.3% and most of the recurrent tumors were diagnosed within 2 years after TURBT (Fig. 5). Based on this observation, we selected the 2-year RFS rate after TURBT as the primary endpoint of this trial. In previous clinical trials, the 2-year RFS survival rate after TURBT with IPIC ranged from 65 to 85% in patients with low- and intermediate-risk NMIBC [18, 24–26].

The required sample size was determined to test our null hypothesis of superiority in 2-year RFS rate after surgery. We hypothesized that PDD-EBTUR will suppress the risk of recurrence by 75%, yielding a 2-year RFS of 80% in the PDD-cTURBT group and by 95% in the PDD-EBTUR group. We calculated the sample size using EZR 1.37 (Saitama Medical Center, Jichi Medical University, 2018). To provide 80% power (β = 0.20) and an α level of 0.05 (two-sided), 48 patients are required in each arm with 3-year registration period and 2-year tracking period years. Assuming that 70% of patients would be diagnosed pathologically with Ta low-grade tumor without concomitant CIS and that the rate of dropout before the completion of the two-year follow-up would be approximately 10%, we plan to enroll 80 patients initially in each arm. Therefore, a total of 160 patients with clinically Ta-T1N0M0 bladder cancer will be enrolled and allocated by computer-aided randomization to either the PDD-cTURBT or PDD-EBTUR group (Fig. 1). Target sample size would be reached according to our annual record of surgery.

### Interim analysis and monitoring

Because this is a short-term study with a 2-year follow-up period, we will not conduct interim analysis. However, the safety of PDD-EBTUR will be independently evaluated by the Safety Monitoring Committee when:

i) A critical modification of the study protocol is required
ii) Any serious adverse event associated with this surgical procedure occurs
iii) A critical problem is observed while monitoring
iv) The principal investigator needs the judgment of this committee

Protocol amendments based on the judgment of Safety Monitoring Committee, regarding eligibility criteria, outcomes, and analyses, will be released to relevant parties (investigators, the Ethics Committee of Nara Medical University, trial participants, and trial registry).

Principal Investigator reports about implementation of the clinical trial, data correction, adverse events, and protocol deviation and violation to the data monitoring committee (DMC), at least when the first patient is enrolled and annually. The DMC is composed by the Clinical Research Center of Nara Medical University, which evaluate whether the study is implemented in compliance with the study protocol and whether the data are appropriately corrected according to a pre-arranged monitoring plan, independently from the clinical trial team. The DMC has no potential conflict of interest. The Trial Steering Committee was not set up in this clinical trial.

### Statistical analysis

The full analysis set (FAS) population will be analyzed for both the primary endpoint (2-year RFS rate) and survival outcomes (Fig. 3; RFS after 2 years, progression-free survival, progression-free rate, upper urinary tract cancer-free survival, metastasis-free survival, cancer-specific survival, and overall survival), and the per protocol set (PPS) population will be analyzed for the other secondary endpoints. A two-sided *P* value of less than 0.05 will be considered to be statistically significant.

To compare the PDD-cTURBT and PDD-EBTUR groups, descriptive statistical analysis will be performed for all study variables. To assess the prognostic impact of the surgical procedure and clinicopathological parameters, event-free survivals will be estimated using the computing risks model; this model considers patients who die of other causes (competing events) before recurrence or progression. The Gray test and Fine-Gray subdistribution hazard regression will be applied to the primary endpoint and the survival outcomes at the secondary endpoints to compare the groups and to calculate the hazard ratio. In addition, a sensitivity analysis of the primary endpoint will be performed using the log-rank test and Cox proportional hazard regression analysis. The amount of change in other post-operative observations over time will be analyzed using the repeated measures analysis of variance (ANOVA), Wilcoxon signed-rank sum test, or Mann-Whitney *U* test. For analyses of cross-sectional measures for continuous and categorical variables, the Mann-Whitney *U* test and Fisher’s exact test, respectively, will be used. A subgroup analysis will be performed to examine whether effects of the intervention differ between subgroups according to the characteristics of patients.

### Dissemination

The results will be submitted to a peer-reviewed journal for publication and will be presented at local and international scientific conferences. Additionally, the trial results will be made available for interested participants.

## Discussion

A systematic review and meta-analysis revealed that EBTUR was superior to cTURBT in terms of 2-year recurrence-free rate, complication rate, hospitalization time, and catheterization time [10]. However, only one RCT was included in the aforementioned review. We launched this RCT because there is a significant need for more consolidated evidence. In this RCT, PDD-EBTUR will be performed by a single surgeon using a predefined surgical technique described in our previous study [15]. Based on our initial experience, we believe that PDD-EBTUR is a simple, easy, safe, and acceptable surgical method. To the best of our knowledge, this is the first study conducted to compare the clinical benefits of oral PDD-EBTUR and PDD-cTURBT.

We previously established risk tables for the current clinical setting, which enabled short- and long-term risk stratification for intravesical recurrence, progression, and cancer-specific death after TURBT in NMIBC [17]. The risk table for intravesical recurrence consisted of five factors: age (20–69 years old or 70–85 years old), past history of NMIBC (primary or recurrent), multiplicity (solitary or multiple), tumor size (cutoff: 30 mm), and T category of TUR specimens (Ta or T1). In this trial, tumors > 30 mm in size do not meet the inclusion criteria, and TUR specimens categorized as T1 will be excluded from primary endpoint analysis. The remaining three factors will be included as factors for stratification. According to the systematic review by Yang et al. [14], the mean size of bladder tumors treated by EBTUR seemed to be approximately 20 mm, which implies that a tumor size of approximately 20 mm is suitable and reasonable for EBTUR. The opinion of M. Miyake, an experienced EBTUR surgeon, is that tumors that are more than 20 mm in size usually require longer operation time and great skill compared to smaller tumors. Therefore, we decided that tumors of size 6–20 mm or 21–30 mm should be selected as an allocation factor. Tumors that are less than 6 mm in size will not be included because small tumors can be resected with a single cut using a standard loop electrode. As a result, four factors will be considered during participant randomization into the PDD-cTURBT or PDD-EBTUR group.

Functional patient-reported outcomes after TURBT, especially EBTUR, have been rarely investigated. Maximal effort should be made to mitigate postoperative urinary symptoms and deterioration of the QOL. A previous study demonstrated that patients with NMIBC are affected psychologically during the period between initial diagnosis using cystoscopy and TURBT [27]. Our previous report focused on the difference in the worsening of urinary symptoms and deterioration of health-related QOL between the EBTUR and cTURBT groups. The score of the daytime frequency (IPSS 2) increased only in the EBTUR group, but not in the cTURBT group [15]. We speculated that the resection of deeper layers of the bladder wall, the wider range of the bladder mucosa, and the longer operative time for the EBTUR group compared to the cTURBT group might be associated with the difference in the worsening of urinary symptoms. Moreover, QOL assessment revealed a decrease in emotional and mental scores among patients in the EBTUR group but not among patients in the cTURBT group. Presently, we cannot draw a conclusion regarding the difference between EBTUR and cTURBT. We plan to perform multiple assessments of the functional outcomes. Therefore, the present RCT will elucidate the true clinical value of EBTUR.

Successful management of NMIBC depends on complete resection and accurate pathological diagnosis. The goal of this prospective, single-center RCT is to determine the potential benefit of PDD-EBTUR followed by IPIC in patients with low- or intermediate-risk NMIBC who undergo TURBT. Ultimately, our findings will lead to the development of better interventions and potentially change the standard of care.

## Trial status

The study began in August 2020. As of February 2021, patient recruitment has not yet been completed and the intervention program is ongoing. Patient recruitment is expected to be completed in July 2023. Follow-up and data collection will be completed in July 2025. The final results are expected in December 2025.

## Supplementary Information

**Additional file 1.** SPIRIT 2013 Checklist for the FLEBER study.

**Additional file 2.** The consent form given to participants.

**Additional file 3. **The explanatory document translated to English (the original document is written in Japanese).

## Data Availability

The collected datasets used during this clinical trial are available from the corresponding author (M. Miyake) on reasonable request such as email request to the lead author for the purpose of systematic review and meta-analysis.

## Abbreviations

TURBT: Transurethral resection of bladder tumor
NMIBC: Non-muscle invasive bladder cancer
RCT: Randomized controlled trial
ALA: 5-aminolevulinic acid
PDD: Photodynamic diagnosis
EBTUR: En bloc transurethral resection of bladder tumor
cTURBT: Conventional transurethral resection of bladder tumor
HR-QOL: Health-related quality of life
CIS: Carcinoma in situ
BCG: Bacillus Calmette-Guérin
IPIC: Single immediate postoperative intravesical instillation of chemotherapy
PRO: Patient-reported outcome
IPSS: International Prostate Symptom Score
OABSS: Overactive bladder symptom score
AE: Adverse event
CTCAE: Common Toxicity Criteria for Adverse Events
