# Supplementary material for: Fluorescent cystoscopy-assisted en bloc transurethral resection versus conventional transurethral resection in patients with non-muscle invasive bladder cancer: study protocol of a prospective, open-label, randomized control trial (the FLEBER study)
Source: Trials. 2021 Feb 12;22:136. doi: 10.1186/s13063-021-05094-y (PMC7881486; doi:10.1186/s13063-021-05094-y)
Supplement: Supplementary file 2 — Additional file 2. The consent form given to participants. [file 13063_2021_5094_MOESM2_ESM.pdf]

Consent form

Nara Medical University President, Yuji Hosoi

Name of trial : The FLEBER study

Fluorescent cystoscopy-assisted en bloc transurethral resection versus conventional transurethral resection in patients with non-muscle invasive bladder cancer

Participants should a tick box when they receive explanation and understand each item.

【Explanation】

- ☐ Regarding this clinical trial
- ☐ Research organization for this clinical trial
- ☐ Reasons for being selected as a subject of clinical research
  - ➢ About your disease and surgery
  - ➢ Purpose of this clinical trial
  - ➢ Criteria for participation in this clinical trial
  - ➢ Treatment after the end of clinical trial
  - ➢ Handling of results after the end of clinical trial
- ☐ Expected benefits and possible disadvantages
- ☐ Participation in and withdrawal from this clinical trial
- ☐ If you want to withdraw your participation in this clinical trial
- ☐ There is no disadvantage to refusing to participate in this clinical trial or withdrawal
- ☐ Disclosure of information on this clinical trial
- ☐ Regarding materials related to this clinical trial
- ☐ Regarding privacy protection
- ☐ Storage and usage of samples / information and storage period
- ☐ Secondary use of data obtained in this clinical trial
- ☐ Funding sources and conflicts of interest
- ☐ Contact for inquiries regarding this trial
- ☐ Regarding the cost of clinical research
- ☐ Other treatments for your disease
- ☐ When this clinical trial causes health hazards
- ☐ Regarding the Medical Ethics Review Committee
- ☐ What I want you to protect
- ☐ After the trial is completed, the data and information should be saved and anonymized for use in the possible future resarch

【Participant's signature】

In participating in this research, I have received sufficient explanations about the above matters, received explanatory documents, and fully understood the contents, so I agree to participate in this trial. We have confirmed that this consent can be withdrawn at any time.

Agreement date : 20 / / Signature : ______________________

Adress : ______________________________ Birth : ____/____/____

【Signature of medical doctor or research collaborator】

I (we) fully explained the clinical research using explanatory documents regarding the above explanations.

Explanation date : 20 / / Affiliation : ____________ Signature : ______________
