# Supplementary material for: Fluorescent cystoscopy-assisted en bloc transurethral resection versus conventional transurethral resection in patients with non-muscle invasive bladder cancer: study protocol of a prospective, open-label, randomized control trial (the FLEBER study)
Source: Trials. 2021 Feb 12;22:136. doi: 10.1186/s13063-021-05094-y (PMC7881486; doi:10.1186/s13063-021-05094-y)
Supplement: Supplementary file 3 — Additional file 3. The explanatory document translated to English (the original document is written in Japanese). [file 13063_2021_5094_MOESM3_ESM.pdf]

Explanatory document

「Fluorescent cystoscopy-assisted en bloc transurethral resection versus conventional transurethral resection in patients with non-muscle invasive bladder cancer: study protocol of a randomized controlled trial (the FLEBER study)」

Nara Medial University

July, 31st, 2020 Approved

0. Introduction

It is the mission of university hospitals to establish new treatments through clinical research, which can be achieved with the cooperation of patients. It is planned and planned by a doctor who is involved in actual medical care based on the medical necessity and importance.

This booklet is a document that describes clinical research. Please read this document carefully and consider whether you agree to participate in the clinical study. If you have any questions about this documentation, feel free to ask your doctor.

This clinical study is in the stage of examining the "verification of efficacy" of "transurethral resection of bladder tumor with fluorescent cystoscope", which is one of the treatments for bladder cancer.

1. Regarding this clinical trial

The clinical study we ask for your cooperation this time is a trial called "Randomized controlled trial (FLEBER trial) for transurethral tumor mass resection and conventional resection using fluorescent cystoscope for nonmuscular layer invasive bladder cancer". ..

In clinical research conducted at Nara Medical University, the medical ethics review committee examines whether there are any problems with the protection of human rights and safety of those who participate in the research and the scientific nature, and based on that opinion, the president Only those approved by will be implemented.

The outline of this research is registered in the public database / University Hospital Medical Information Network (UMIN), and the research plan is changed and the progress of the research is updated as appropriate (number: UMIN000041273).

The clinical study will be conducted from the date of publication of the implementation plan to December 31, 2025, with 160 patients scheduled to participate. If a patient participates in this study, the planned participation period is approximately 1 week to 10 days in the hospital and approximately 2 years in the outpatient follow-up period.

2. Research organization

Principal Investigator:

Nara Medical University Hospital, Department of Urology, Lecturer, Makito Miyake

Investigators:

Nara Medical University Hospital, Department of Urology, Kiyohide Fujimoto

Kazumasa Torimoto, Satoshi Anai, Yasushi Nakai, Shunta Hori

3. Reasons for being selected as a subject of clinical research

A, About your disease

The disease you currently have is a metastatic bladder tumor (bladder cancer). The risk factors for bladder cancer are elderly / male and smoking history, and the number of patients is increasing with aging.

For bladder cancer, the T stage is determined by the depth of the root, as shown in the figure above. Tis, Ta, and T1 are called non-muscle invasive bladder cancer because their roots do not extend to the muscular layer. On the other hand, if the root extends to the muscle, it is called muscular invasive cancer (T2 or higher in the above figure), and surgery called "total cystectomy" is required to remove the entire bladder. And at present, your bladder cancer is classified as the former non-muscle invasive bladder cancer, and one of the surgical treatments, "fluorescent cystoscopic transurinary bladder tumor resection" is planned.

Most non-muscle invasive bladder cancers do not require total cystectomy and can be treated with transurethral resection of the bladder tumor. The problem is that the tumor may come back in the bladder after resection. This is called bladder recurrence. If bladder recurrence is found, readmission and reoperation will be required. Therefore, how to prevent this bladder recurrence is a big issue. Since the course of this disease varies after surgery, it is classified into four categories according to the risk (risk): (1) low risk, (2) medium risk, (3) high risk, and (4) highest risk. In the case of high-risk or highest-risk, the disease may progress to muscular invasive

cancer (T2 or higher) without treatment, so transurethral surgery may be performed again or bovis-type tuberculosis bacterium. Treatment is added by injecting a drug called BCG into the bladder. On the other hand, in low-risk or medium-risk cases, an anticancer drug is injected into the bladder immediately after transurethral resection of the bladder to prevent recurrence. Even with these treatments, they may recur in the bladder. Looking at the outcomes of patients we have treated so far, we find that in low to medium risk cases, about 20% of patients have bladder recurrence within 2 years after surgery.

One of the causes of recurrence is that tumor cells are scattered and scattered in the bladder during transurethral resection of the bladder tumor. Therefore, it is considered important to devise as much as possible to prevent tumor cells from being scattered during surgery.

B, Difference between the conventional surgical method and the new method

Tumors need to be removed from the body through the urethra. Most tumors that form in the bladder are thicker than the urethra, and traditional methods shred the tumor and remove it from the body. Since this method is simple, it has been used for a long time. However, it is inevitable that the tumor will spread into the bladder during shredding. To prevent this, we have recently tried a new surgical method called "transurethral resection of bladder tumor".

It is hoped that this method will prevent tumor cells from spreading into the bladder during surgery and reduce the recurrence of the bladder later. However, it takes a little longer to remove the tumor as a group compared to the conventional method, and if the tumor is too large (eg, a tumor larger than 30 mm), it is pulled out of the urethra. It also has the disadvantage of not being able to. The current situation is that it has not yet been verified which of the conventional treatment and the new treatment is really good for the patient.

C, Purpose of this clinical trial

In this clinical study, patients undergoing transurethral resection of bladder tumors for bladder cancer were randomly assigned to two groups, and either conventional or new surgery was performed. The purpose is to receive it and investigate the effectiveness and safety of each.

D, Criteria for participation in this clinical trial

There are some conditions for participation in this research. The doctor in charge will make a comprehensive judgment based on the results of medical examinations and various tests, so please ask the doctor in charge for details. Therefore, depending on the results, you may not be able to participate in this research.

☞ Participants: (Those who meet all of the following conditions)

- Those who undergo fluorescent cystoscopic transurethral resection of bladder tumor for bladder cancer

- Tumor major axis is 6 mm or more and 30 mm or less

- Non-muscle invasive bladder cancer: The T stage mentioned above is Ta or T1.

- Age 20 years old and over 85 years old and under

- Good general condition: You can do your own personal belongings and spend more than 50% of the day out of bed.

- As a result of blood test, bone marrow function and organ function are leaning.

  Hemoglobin: 9.0 g / dL or more

  White blood cell count: Facility standard value lower limit ~ 12,000 / mm3 or less

  Number of neutrophils: 2,000 / mm3 or more

  Platelet count: 100,000 / mm3 or higher

  Total serum bilirubin level: less than twice the upper limit of the facility standard value

☞ Those who cannot participate: (Those who meet any one of the following conditions)

- Patients with suspected presence of carcinoma in situ (Tis)

- Patients with a history of hypersensitivity to 5-aminolevulinic acid salt

- Patients with hepatic dysfunction / inflammatory disease

- Patients with porphyria

- Patients who cannot consent to participate in the study on their own initiative (However, even if the explanation can be understood and agreed, if it is difficult for the patient to sign due to limb disorders, etc., the patient's consent The author may sign the confirmation.)

- Patients who are judged by the investigator or investigator to be inappropriate for participation in this clinical trial

2. Flow chart of this clinical trial

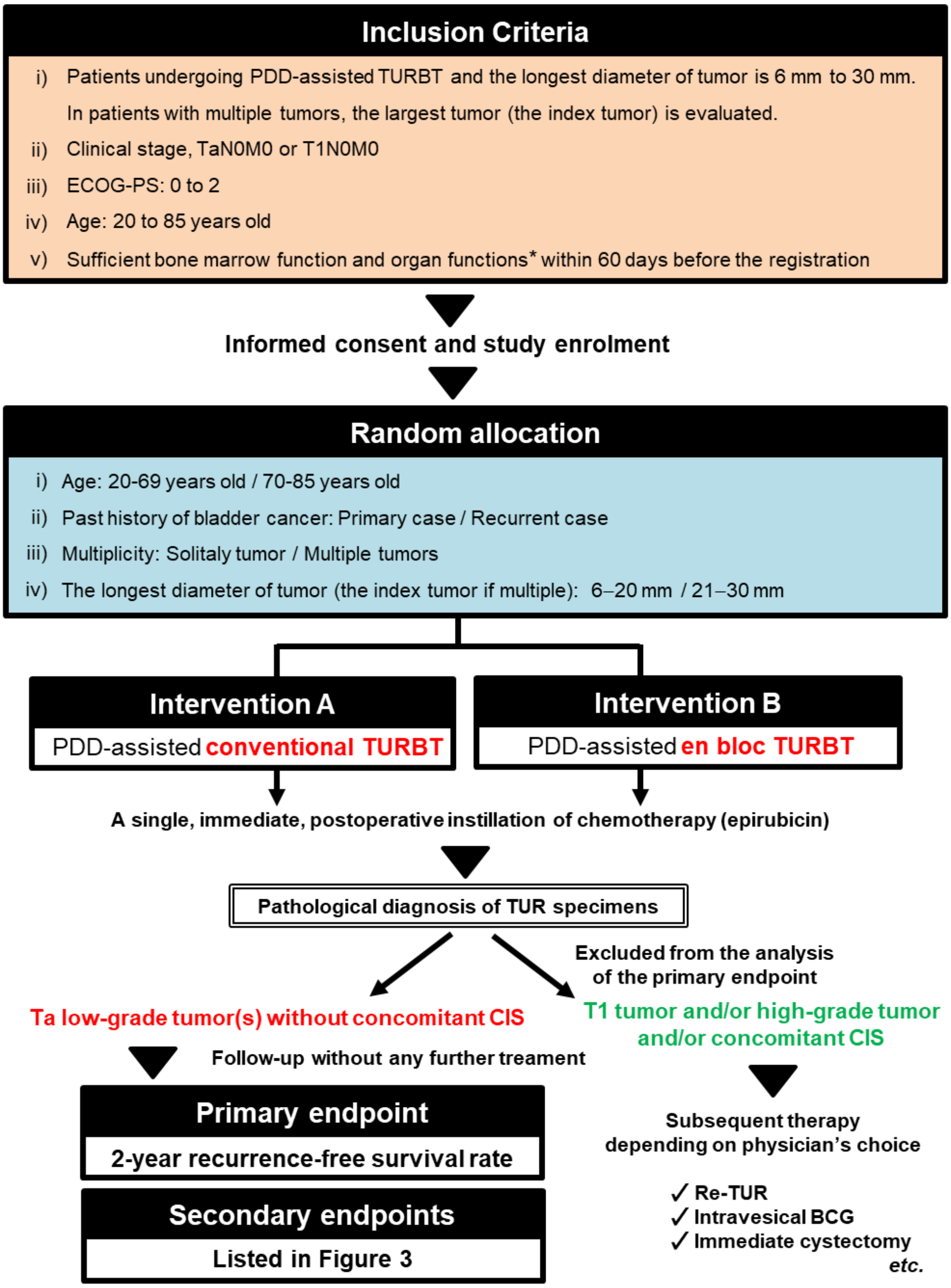

The flow of this clinical study is shown above. If you agree to this study, we will assign you to either Group A: standard treatment / control group or Group B: new treatment / trial group based on four predetermined allocation factors, and perform surgery. You will receive it. After that, you will be examined and examined according to the schedule shown below. As shown in the schedule below, you will be asked to cooperate with the record of the urination diary at 4 points x 3 days, and a total of 4 questionnaires on quality of life and pain. The study will end about two years after the surgery, but even after the study period ends, regular visits must be continued to confirm the recurrence of the cancer. In addition, if your doctor deems it necessary, you may be asked to undergo additional tests and examinations depending on your health condition.

	STUDY PERIOD													
	Screening Phase (Baseline)	Hospital admission	Surgery	Hospital discharge	Follow-up visits after surgery									....
TIMEPOINT	$-t_{60}$ to $-t_1$	$-t_2$ to $-t_1$	0	t_4 to t_{10}	1 month	3 months	6 months	9 months	12 months	15 month	18 months	21 months	24 months	....
ENROLMENT :														
Eligibility screen	X													
Informed Consent	X													
Physical Exam (Height, BW, BT, BP)	X													
ECOG-PS	X													
Complete Blood Count *	X		X	X	X	X	X							
Serum chemistry **	X		X	X	X	X	X							
Urianalysis ***	X				X	X	X	X	X	X	X	X	X	X
Chest-abdomin-pelvis CT and /or MRI	X													
Randomization	X													
INTERVENTION :														
PDD-cTURBT or PDD-EBTUR			X											
ASSESSMENTS :														
Cystoscopy	X					X	X	X	X	X	X	X	X	X
Urine cytology	X	X			X	X	X	X	X	X	X	X	X	X
Postvoid residual volume (Portable Bladder Scanner)		X			X	X	X							
Frequency volume chart †		X			X	X	X							
Pain scales††				X	X	X	X							
Adverse events †††					X	X	X	X	X	X	X	X	X	
Health-related QOL questionnaire ††††		X			X	X	X	X	X	X	X	X	X	
IPSS and OABSS questionnaire		X			X	X	X	X	X	X	X	X	X	
Follow-up (checking survival)					X	X	X	X	X	X	X	X	X	X

Follow-up visits and data collection should occur approximately 1, 3, 6, 9, 12, 15, 18, 21, and 24 months from the surgery. Patients will complete a set of questionnaires at every visit, and follow-up information may be collected via medical charts. The Case Report Form will include information regarding past history, concomitant medications, and any medications taken after the treatment. Chest–abdomen–pelvis computed tomography (CT) and/or magnetic resonance imaging (MRI) should be performed for TNM classification. X, mandatory; * Hemoglobin, hematocrit, white blood cell count and fractions, platelet count; ** aspartate transaminase (AST), alanine transaminase (ALT), □-glutamyl transpeptidase (□-GTP), total bilirubin, alkaline phosphatase (ALP), lactate dehydrogenase (LDH), total protein, albumin, serum creatinine, uric acid, total cholesterol, low-density lipoprotein (LDL)-cholesterol, high-density lipoprotein (HDL)-cholesterol, triglyceride, C-reactive protein (CRP), calcium, phosphorus, potassium, chloride; *** Urine dipstick test (specific gravity, pH, protein, glucose, bilirubin, urobilinogen, ketone body, and occult blood) and urine sediment test; † 3 days and nights record. Patients pick days that will be convenient for them to measure and record everything; †† Pain scale measures: Numerical Pain Rating Scale (NPRS), Visual Analogue Scale (VAS), and Faces Pain Scale (FPS); †††According to the Common Toxicity Criteria for Adverse Events (CTCAE v 5.0) translated into Japanese; †††† Questionnaires: SF-8□, EORTC QLQ-C30, and FACT-BL. Abbreviations: BW, body weight; BT, body temperature; BP, blood pressure; ECOG-PS, Eastern Cooperative Oncology Group-performance status scale; PDD, photodynamic diagnosis; EBTUR, en bloc transurethral resection of bladder tumor; cTURBT, conventional transurethral resection of bladder tumor; QOL, quality of life; IPSS, International Prostate Symptom Score; OABSS, overactive bladder symptom score.)

About the planned number of cases and research period

(1) Scheduled number of cases: 160 cases (expected to register 80 cases per year)

(2) Research period: Implementation approval date-December 2025
(Registration period: ~ July 2023, Follow-up period: ~ July 2025, Analysis period: ~ December 2025)

Treatment after the end of clinical research

No particular treatment has been decided after the clinical study is completed. Even after the end of the study period, regular visits will continue to confirm the recurrence of the cancer.

Handling of results after the end of clinical research

The rights related to intellectual property such as patents generated as a result of this clinical study belong to the Department of Urology, Nara Medical University.

3. Expected benefits and possible disadvantages

- **Expected benefits (effects)**: All surgical treatments and drugs used in this clinical study are approved for indication and covered by insurance for bladder cancer, which is the target disease, and all treatment methods in any group can be performed as daily insurance medical treatment. is. In addition, all medical expenses including drug costs during the patient's study period are paid by the patient's insurance and self-pay, so compared to daily medical care, the special benefits that patients can obtain by participating in this study. There is no medical or financial benefit. In addition, blood tests, urinalysis, and diagnostic imaging tests required for follow-up after surgical treatment are also performed within the scope of normal insurance medical care, so this point is also a special medical care for patients participating in this study. There is no top or financial benefit. It has been suggested that the risk of bladder recurrence is reduced as a benefit of tumor mass resection performed in the new treatment / trial group of group B. Based on these previous studies, if he were assigned to Group B, he could benefit from a reduced risk of bladder recurrence. However, the current situation is that it has not yet been verified which is really better for the patient, conventional surgery or new surgery.

- **Expected disadvantages:** Transurethral resection of bladder tumor with fluorescent cystoscope and single intravesical infusion therapy of anticancer drug after surgery, which are performed in both groups A and B, are performed as usual insurance medical care and are routine medical care. We do not believe that there will be any special dangers or disadvantages compared to. We believe that there is no risk of complications added in group B or any increased disadvantages from participating in this study. According to past reports conducted in Japan, the complications observed after undergoing conventional fluorescent cystoscopic transurethral resection of the bladder tumor were found in 58 of 61 patients (95%). No adverse events have been observed, but no life-threatening complications have been observed. There were a few cases in which the discharge period was postponed or special treatment was required, and blood ALT concentration increased (4 cases), blood glucose level increased (1 case), bladder perforation (1 case), and hypotension (1 case).), Urticaria (1 case), etc. The risk of these complications is not increased by participating in this study, but is a well-known complication that is also caused by the surgery and treatment that we generally perform. Therefore, it is unlikely that the frequency of complications will increase regardless of whether they are assigned to Group A or Group B. To ensure that you participate in the study safely, your doctor will pay attention to your physical condition and test values to see if there are any such complications. If such unfavorable complications are found during the study, a specialist doctor will provide appropriate treatment. In addition, at the discretion of the doctor in charge, oral medication may be prescribed or the study may be discontinued.

4. Participation in and withdrawal from clinical research

After hearing the explanation of this clinical study from your doctor, you are free to decide whether or not to participate. If you decline to participate, you will not be penalized. As always, your doctor will give you the best possible treatment. In that case, the doctor in charge will decide whether to receive treatment with conventional surgery or new surgery.

In addition, if new information is obtained during the clinical research that may be related to your decision to continue the clinical research, we will promptly notify you and confirm whether you can continue to participate in the clinical research.

5. If you want to quit your participation in clinical research

You can withdraw at any time after you have participated in this clinical study. If you would like to quit your participation, please contact your doctor. In that case, please describe it in the prescribed consent withdrawal form. If you quit your participation prematurely, you will not be penalized for treatment. As always, your doctor will give you the best possible treatment.

6. Disclosure of information on clinical research

The outline of this research (name, purpose, method, implementation system, etc. of the study) is registered in the public database / University Hospital Medical Information Network (UMIN), and the research plan is changed and updated as appropriate according to the progress of the research. (Registration number: UMIN000041273). Information that identifies the individual patient is not registered.

7. Regarding materials related to clinical research

If you wish, you can browse materials related to research plans and research methods. If you wish, please contact your doctor or the Bioethics Supervision Office. At that time, please note that you may be required to show your driver's license, etc. to verify your identity, and to charge a handling fee.

8. About privacy protection

The materials and information (symptoms, test results, questionnaires, etc.) collected in this clinical study should be anonymized for personal information such as your name and identity (remove descriptions that can identify a specific individual).) And use it. We will give due consideration to the handling of patient information and strictly manage it so that it will not be leaked to the outside. In this study, we use a correspondence table so that individuals can be identified when necessary.

The results of this clinical study may be published in academic societies and medical journals, but since they are anonymized, personal information such as the patient's name and identity will be revealed at that time. It is not. In addition, medical personnel are required by law to keep the confidentiality of patients, and since we handle your information in accordance with the Personal Information Protection Law of Incorporated Administrative Agencies, etc. based on the laws and regulations related to personal information protection, individuals No information is leaked to the

outside. As a personal information manager, Nara Medical University, Department of Urology, Lecturer on campus, Makito Miyake will be in charge.

In addition, in order to confirm whether the clinical research is being carried out properly, the persons involved in this clinical research (such as the staff of this hospital), the person in charge of monitoring who is not directly related to this clinical research, and the medical ethics review Committee members may look at your medical records, but these people are obliged to keep secrets about what they know at work and work under the Personal Information Protection Act, so your privacy is There is no need to worry about leaking to the outside. If you agree to participate in the research, you also acknowledge that the above persons will see the contents of the medical records.

9. Storage and usage of samples and information, and storage period

The materials and information provided will be provided to our data center, but they will be collected in a way that only those involved in this research can access, and will be stored for 5 years after the end of the research. The materials and information provided will be provided to researchers after a fair examination of the research content and will never be bought or sold. No compensation will be paid to you who provided the materials. After the research is completed (when the storage period is over), the samples and information will be discarded as they are processed so as not to identify individuals.

We may conduct new research using the materials and information provided in this research (called secondary use of data). In this case, the research will be conducted again through the necessary procedures such as receiving appropriate examination by the clinical research examination committee of Nara Medical University and the medical ethics examination committee, and obtaining the president's permission. I will do it. The contents of these studies will be posted in "Clinical Research" in the right column of the homepage "http://www.naramed-u.ac.jp/~urol/" of the Department of Urology, Nara Medical University. We are here. Please contact us if you do not want to use it for other studies.

10. Research funding sources and conflicts of interest

In conducting clinical research, a third party may suspect that fair and proper judgment will be impaired for the benefit of the company. Such a situation is called a "possible conflict of interest (conflict of interest)". Possible conditions that hinder fair and proper judgment include falsification of data, preferential treatment of specific companies, and conditions in which research should be stopped but continued. All medical treatment in this clinical study is covered by insurance, so no special funds are required. For miscellaneous expenses, we will use our own funds from the Department of Urology, Nara Medical University. Therefore, this clinical study has no relationship with companies.

11. Consultation for this research

If you have any concerns or questions regarding this clinical study, please do not hesitate to contact your doctor or the consultation desk. If you need other treatment due to illness or injury, please contact your doctor or the consultation desk.

Department of Urology, Nara Medical University Hospital

Telephone: 0744-22-3051 (extension: 2338)

12. About the cost of clinical research

If you participate in this study, there will be no increase in blood tests or physical examinations for this study. Treatment and follow-up for bladder cancer are performed within the scope of normal insurance medical treatment. Expenses related to medical treatment (out-of-pocket expenses for regular insurance medical treatment: medical fees, drug costs, part of examination costs, etc.) will be borne by the patient as in the past as normal insurance medical treatment. However, the urination diary related to this study (record the time of urination and the amount of urination in one day: records before surgery and at 1, 3 and 6 months after treatment. In principle, the number of days recorded at each time point is 3 days. Minutes. Use the special recording paper and plastic cup with memory prepared here. A photo is attached on page 19 of this

document), a questionnaire on quality of life and pain (the time required for the questionnaire is about 10 minutes). It is necessary to describe such items separately from the regular medical treatment. The research group will cover these costs. In addition, if you need to receive medical treatment for a disease unrelated to the content of your research during the research period, you will be required to pay your own expenses through regular insurance medical treatment. Please understand that there are no special rewards for participating in this research.

13. When clinical research causes health hazards

Patients participating in this clinical trial may suffer health hazards from complications and side effects as described above in “Expected Disadvantages”. We carefully plan this clinical study to reduce those possibilities and strive to minimize the patient's disadvantages during the clinical study, but these disadvantages are You can't eliminate all the possibilities. In the unlikely event that this clinical study causes health hazards such as unpredictable serious side effects, we will take appropriate measures according to the medical condition as in normal medical care, but medical expenses equivalent to the self-pay portion. You will have to pay for it yourself. This clinical study does not provide any special financial compensation such as condolence money or various allowances.

14. What I want you to protect

Please inform your doctor if you start a new drug, change the drug you are taking, or visit another hospital during the study period. Also, please note that if you are visiting another hospital, we may notify that hospital that you are participating in this clinical study when you receive an inquiry.

In the following cases, even if you intend to continue clinical research, we may cancel it at the discretion of your doctor. Even in that case, the doctor in charge will give the best treatment.

- When it is found that the conditions for participation in this clinical study are not met
- When it is necessary to change the treatment method depending on the condition of the disease
- When the entire study is canceled
